# Keeping the Wolves at Bay: Antitoxins of Prokaryotic Type II Toxin-Antitoxin Systems

**DOI:** 10.3389/fmolb.2016.00009

**Published:** 2016-03-22

**Authors:** Wai Ting Chan, Manuel Espinosa, Chew Chieng Yeo

**Affiliations:** ^1^Molecular Microbiology and Infection Biology, Centro de Investigaciones Biológicas, Consejo Superior de Investigaciones CientíficasMadrid, Spain; ^2^Faculty of Medicine, Biomedical Research Centre, Universiti Sultan Zainal AbidinKuala Terengganu, Malaysia

**Keywords:** toxin-antitoxin, DNA-binding motifs, transcriptional repressor proteins, autoregulation, conditional cooperativity

## Abstract

In their initial stages of discovery, prokaryotic toxin-antitoxin (TA) systems were confined to bacterial plasmids where they function to mediate the maintenance and stability of usually low- to medium-copy number plasmids through the post-segregational killing of any plasmid-free daughter cells that developed. Their eventual discovery as nearly ubiquitous and repetitive elements in bacterial chromosomes led to a wealth of knowledge and scientific debate as to their diversity and functionality in the prokaryotic lifestyle. Currently categorized into six different types designated types I–VI, type II TA systems are the best characterized. These generally comprised of two genes encoding a proteic toxin and its corresponding proteic antitoxin, respectively. Under normal growth conditions, the stable toxin is prevented from exerting its lethal effect through tight binding with the less stable antitoxin partner, forming a non-lethal TA protein complex. Besides binding with its cognate toxin, the antitoxin also plays a role in regulating the expression of the type II TA operon by binding to the operator site, thereby repressing transcription from the TA promoter. In most cases, full repression is observed in the presence of the TA complex as binding of the toxin enhances the DNA binding capability of the antitoxin. TA systems have been implicated in a gamut of prokaryotic cellular functions such as being mediators of programmed cell death as well as persistence or dormancy, biofilm formation, as defensive weapons against bacteriophage infections and as virulence factors in pathogenic bacteria. It is thus apparent that these antitoxins, as DNA-binding proteins, play an essential role in modulating the prokaryotic lifestyle whilst at the same time preventing the lethal action of the toxins under normal growth conditions, i.e., keeping the proverbial wolves at bay. In this review, we will cover the diversity and characteristics of various type II TA antitoxins. We shall also look into some interesting deviations from the canonical type II TA systems such as tripartite TA systems where the regulatory role is played by a third party protein and not the antitoxin, and a unique TA system encoding a single protein with both toxin as well as antitoxin domains.

## Introduction

The profusion of toxin-antitoxin (TA) genes among the realm of prokaryotes has sparked the interest of researchers to reveal the rationale of TA existence. One could hardly imagine the reason behind the finding that TA, which is mainly found in the genomes of bacteria and archaea, can be present up to 88 copies *in Mycobacterium tuberculosis*, although only 30 of them are functional (Ramage et al., 2009). In general (but not in all cases), a TA system is comprised of two genes, the antitoxin gene and its cognate toxin gene, which are located adjacent to each other. There are various modes of action by the toxin protein to exert its toxicity, but the most common ones involve inhibition of translation or replication, or targeting the cell wall synthesis of the host cells. TA systems, which have not been found in eukaryotes are, however, also able to poison eukaryotic cells because eukaryotes share common transcription and translation machineries with prokaryotes (Christensen et al., 2001; Pimentel et al., 2005; Nariya and Inouye, 2008; Amitai et al., 2009; Hurley and Woychik, 2009; Yamaguchi and Inouye, 2009; Agarwal et al., 2010; Dienemann et al., 2011; Castro-Roa et al., 2013; Germain et al., 2013). The product of the antitoxin gene, which can be either RNA or protein, is usually less stable compared to the toxin protein. Depending on the mechanism by which the antitoxin neutralizes the toxin, TAs have been categorized into six different types: (i) Type I, in which the antitoxin mRNA binds to its complementary toxin mRNA to prevent translation of the toxin gene; (ii) Type II, the antitoxin is a protein that forms a stable complex with the toxin protein and blocking the active site of the toxin under normal growth conditions; (iii) Type III, the antitoxin is an RNA with multiple tandem repeats that binds directly to the toxin protein rendering the toxin inactive; (iv) Type IV, the antitoxin protein does not bind to the toxin, but antagonize the toxin effect by competing for binding to the cellular target; and (v) Type V, the antitoxin protein is an RNase that cleaves directly its cognate toxin mRNA (Alonso et al., 2007; Hayes and Van Melderen, 2011; Masuda et al., 2012; Cataudella et al., 2013; Unterholzner et al., 2013; Barbosa et al., 2015). A likely new type of TA system (a possible type VI) was recently discovered in *Caulobater crescentus* where both the SocA antitoxin and SocB toxin are proteins, as in types II and IV TA systems. However, in this case, the SocA antitoxin functions as a ClpXP protease adaptor for the SocB toxin, promoting degradation of the toxin and thereby abolishing its lethality (Aakre et al., 2013; Markovski and Wickner, 2013). Thus, in a type VI TA system, the toxin is the unstable partner whereas in type II TA systems, the antitoxins are the labile partners due to their susceptibility to protease degradation. To date, TA systems belonging to types I and II are the most abundant in prokaryotes with type II TAs being the best characterized (Hayes and Van Melderen, 2011; Unterholzner et al., 2013; Bertram and Schuster, 2014; Hayes and Kêdzierska, 2014).

TA genes, which do not seem to be essential to the host cells (Van Melderen and Saavedra De Bast, 2009; Van Melderen, 2010), have been linked in countless ways to the lifestyle of the bacteria. The function of plasmid-encoded TAs has been commonly recognized as to stabilize the plasmid by a phenomenon denoted as post-segregational killing of the daughter cells that do not inherit its parental plasmid (Jaffe et al., 1985; Gerdes et al., 1986) or “addiction,” as once the cells acquire the TA-encoded plasmid horizontally, the cells are no longer able to survive if they lost that plasmid (Lehnherr and Yarmolinsky, 1995; Hernández-Arriaga et al., 2014). Nevertheless, the chromosomally-encoded TA genes are known to have broader impact to the host cells. Since the consequences of toxin effect can be bactericidal or bacteriostatic, chromosomally-encoded TAs have been related to altruistic cell death or stress response when the cells are under unfavorable circumstances. Altruistic cell death adopted the idea of bacterial cells living as a community, and when under stressful states like scarcity in nutrition, some of the cells will “sacrifice” themselves via triggering of their TA systems, subsequently lysing and releasing nutrients for the rest of their populations' need (Aizenman et al., 1996; Engelberg-Kulka and Glaser, 1999). Of course one could argue that instead of altruism, cannibalism (e.g., in *Bacillus subtilis*; González-Pastor, 2011) or fratricide (e.g., in *Streptococcus pneumoniae*; Eldholm et al., 2009) would more likely had happened for bacteria, which are the more primitive life forms. As activation of most of the toxins leads to cell stasis, the postulation of TAs involving in stress response is more widely accepted (Gerdes et al., 2005). The stress response mediated by TAs was well-demonstrated by the persistence phenomenon observed in *Escherichia coli* and other bacteria. Persister cells refer to a small portion of cells among isogenic antibiotic-sensitive bacterial population that stochastically switch to slow growth (or a quasi-dormant state) leading to multidrug tolerance when exposed to antibiotics (Lewis, 2010). In the persister cells, the increased levels of the signaling nucleotide (p)ppGpp (guanosine pentaphosphate/tetraphosphate) trigger slow growth by activating certain TAs through a regulatory cascade, which is dependent on Lon protease and inorganic polyphosphate (Maisonneuve et al., 2013). There are also other studies that demonstrated the involvement of chromosomally-encoded TAs in biofilm formation, increased survival rate, colonization of new niches, phage abortive infection, maintenance of bacterial mobilomes, virulence of pathogenic bacteria, and as anti-addiction modules (Christensen et al., 2001; Rowe-Magnus et al., 2003; Szekeres et al., 2007; Saavedra De Bast et al., 2008; Harrison et al., 2009; Mine et al., 2009; Hallez et al., 2010; Makarova et al., 2011; Armalyte et al., 2012; Norton and Mulvey, 2012; Cheng et al., 2014). Thus, the diversity of TA systems in prokaryotes is reflected in their diversity of cellular function.

## Antitoxins neutralize the lethality of their cognate toxins and also function as DNA-binding proteins that modulate the prokaryotic lifestyle

Type II TA systems are so far, the best studied of the TA families. Like other systems, Type II TAs are usually comprised of two genes with the antitoxin gene preceding the toxin gene, and with both genes co-transcribed from a single promoter located upstream of the antitoxin gene (Leplae et al., 2011). In general, the two TA genes overlap by a few nucleotides, indicating coupled translation of the two genes. Under normal conditions, the antitoxin protein binds avidly to the toxin protein to safeguard its harmfulness to the cells, as it has also been shown by determination of the three dimensional structures of TA complexes. However, because the antitoxin protein seems to be structurally partially folded (Cherny et al., 2005), it is thus more fragile and susceptible to the degradation by the host proteases (e.g., Lon or Clp); antitoxin cleavage would release the more stable toxin protein to act on its cellular target. Hence, the antitoxin protein needs to be constantly replenished in order to avoid a surfeit of toxin proteins. This explains the organization of the majority of type II TA operons: the antitoxin gene preceding the toxin gene would enable the antitoxin to be transcribed and translated before synthesis of the toxin starts.

Toxins target various cellular structures and essential molecular processes and thus, hinder cellular activities (Hayes and Van Melderen, 2011; Hayes and Kêdzierska, 2014). Majority of class II toxins that have been examined to date act as endoribonucleases and thus, inhibit the translation machinery (Yamaguchi and Inouye, 2009; Yamaguchi et al., 2011). Some of these endoribonucleases, such as the MazF toxin, cleave free mRNA in a sequence-dependent manner (Zhang et al., 2003), whereas other endoribonuclease toxins, such as RelE, target mRNA associated with ribosomes (Pedersen et al., 2003). Some type II toxins interfere with the translation process by other means such as the cleavage of initiator tRNA by the VapC toxins of *Shigella flexneri* and *Salmonella enterica* serovar Typhimurium (Winther and Gerdes, 2011), or phosphorylation of elongation factor EF-Tu by the *E. coli*-encoded HipA toxin and the bacteriophage P1-encoded Doc toxin (Schumacher et al., 2009; Cruz et al., 2014). On the other hand, certain type II toxins (such as CcdB and ParE) affect DNA replication by direct inhibition of gyrase activity, which is required to relieve supercoiling that occurs ahead of the replication fork (Bernard and Couturier, 1992; Yuan et al., 2010). The ζ and PezT toxins blocks cell wall synthesis by phosphorylating peptidoglycan precursors, thereby inhibiting the first step in peptidoglycan synthesis (Mutschler et al., 2011).

Type II antitoxins abrogate the lethality of their cognate toxins through a toxin-binding domain, which is usually natively unstructured until formation of the toxin-antitoxin complex. One of the hallmarks of toxin inactivation is a direct interaction whereby the antitoxin wraps around the toxin and inhibits toxin activity by blocking or masking the toxin active site (Blower et al., 2011; Bøggild et al., 2012; Schureck et al., 2014). For example, the *E. coli*-encoded MazE antitoxin wraps across the surface of the MazF toxin, blocking the active site as well as forcing out the S1-S2 loop that stabilizes the catalytic triad of the toxin (Kamada et al., 2003). In the case of the ζ and PezT toxins, inactivation is due to the respective cognate ε or PezA antitoxin sterically hindering ATP/GTP binding within the toxin (Meinhart et al., 2003; Khoo et al., 2007). The *Caulobacter crescentus*-encoded ParD antitoxin inhibits its cognate ParE toxin by binding as a dimer to a conserved complementary patch at the C-terminus of ParE without inducing conformational changes (Dalton and Crosson, 2010). In contrast, binding of the *E. coli-*encoded RelB antitoxin inhibits the RelE toxin by perturbing the toxin structure, specifically through displacement of a flexible α-helix at the C-terminal that contains the Tyr-87 residue essential for RelE activity (Li et al., 2009). Similarly, the VapB5 antitoxin of *M. tuberculosis* act to prevent its cognate VapC5 toxin from binding Mg^2+^ as a co-factor by reorienting the side-chain of VapC5 Arg-112, locking the VapC5 Glu-57 residue in an unfavorable conformation to bind Mg^2+^ (Miallau et al., 2009). In some TA complexes, the antitoxin binds to the toxin but does not occlude the active site of the toxin. The *E. coli*-encoded HipB antitoxin binds far from the active site of its cognate HipA toxin and functions to inhibit the toxin activity by locking the toxin in an inactive open conformation (Schumacher et al., 2009). Similarly, the HigA antitoxin of *Proteus vulgaris* only makes two regions of contact with the HigB toxin, both of which are distant from the HigB active site. The HigB toxin functions as a ribosome-dependent endoribonuclease and it was proposed that binding of HigA sterically inhibits HigB from interacting with mRNA in the A site of the ribosome (Schureck et al., 2014).

The antitoxin protein is not only the nemesis of its cognate toxin, but also the key factor that regulates transcription of the TA operon. The antitoxin is generally a DNA-binding protein that binds, albeit usually weakly, to the operator of the operon to repress its own transcription; whereas the toxin protein, which does not bind to the DNA upstream of the operon, usually serves as a co-repressor, by binding to the antitoxin protein and changing the conformation of the antitoxin-DNA complex, which lead to further repression (Bertram and Schuster, 2014; Hayes and Kêdzierska, 2014; Kędzierska and Hayes, 2016). In some cases, the molar ratio of antitoxin and toxin has great impact on the formation of the TA complex in terms of stoichiometries (Gelens et al., 2013). More importantly, TA complexes with different stoichiometries have different affinity to the binding of the operator (Overgaard et al., 2008; Garcia-Pino et al., 2010; see below). Therefore, the ratio of the antitoxin and toxin is very crucial to the regulation of the transcription of the TA operon and to determine the lifestyle and fate of the bacterial host.

## DNA-binding domains found in antitoxins: helix-turn-helix (HTH), ribbon-helix-helix (RHH) fold, and SpoVT/AbrB-type

**Figure 1:**
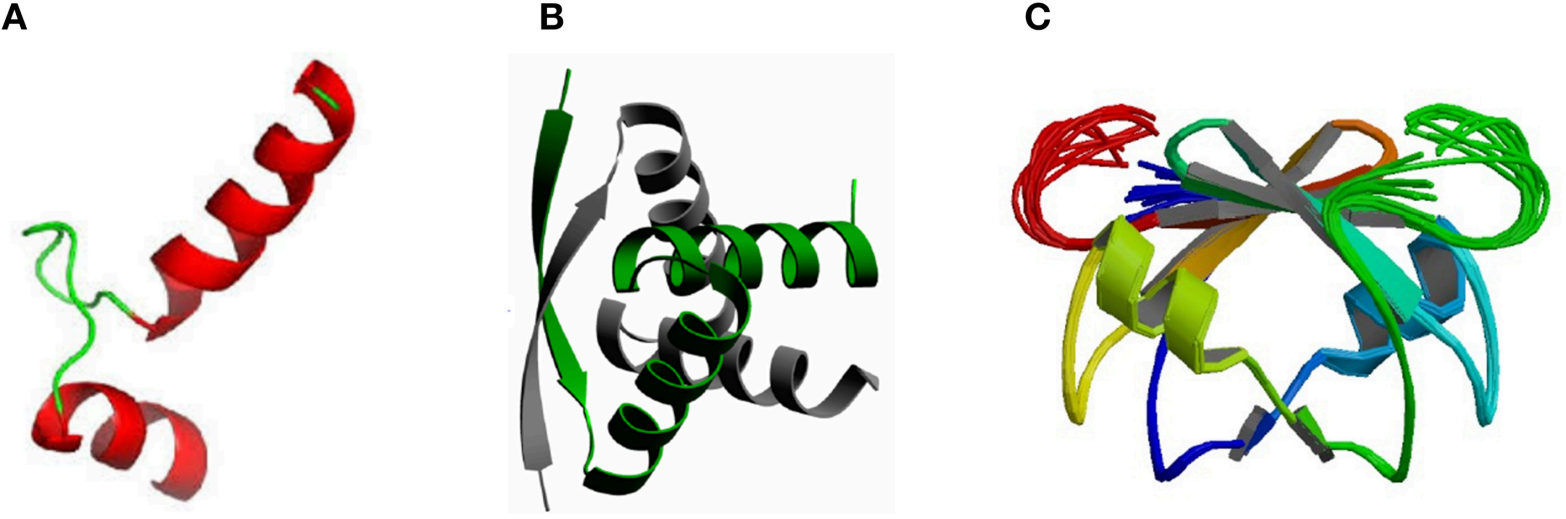
**Three-dimensional structures showing the most frequent DNA-binding domains found in prokaryotic type II antitoxin proteins**. The most frequent DNA-binding domains found in type II antitoxins include: **(A)** the HTH-motif (of which the smallest structural motif is shown) that has two α-helices (red) connected by a small loop (green); **(B)** the RHH folding motif, in which the minimal structure (as the one depicted corresponding to the CopG transcriptional repressor) is generated by two antiparallel β-strands (arrows, with arrowheads pointing to the C-terminal part of the protomer) that generate a ribbon; each strand comes from one of two protein monomers and they are involved both in dimer formation and in specific interactions with the DNA bases in the antitoxin DNA target (adapted from Gomis-Rüth et al., 1998), and **(C)** the SpoVT/Abr DNA binding motif in which the dimeric molecules are constructed by three- and four-stranded antiparallel β-sheets (upper part of the molecule) that are tightly packed, generating the DNA-binding domain. Loops keeping the outer part of the molecules are indicated by light green and red colors.

Concerning the three-dimensional structure of the TAs, several of them have been determined, either the antitoxin alone or in complex with the cognate toxin. In most cases, the antitoxin protein appears to be divided into two domains: the N-terminal domain usually comprises the DNA binding region, whereas the C-terminal domain is generally involved in the interaction with the cognate toxin to offset its toxicity. These two domains may be interconnected by a flexible small loop or hinge-like region. Determination of the crystal structure of the TA complexes (mostly without DNA) has been achieved for an increasing number of them. In general, the structure of the DNA-binding domains of the antitoxins can be grouped into three different types, namely helix-turn-helix (HTH), ribbon-helix-helix (RHH), and SpoVT/AbrB-type (Table 1; Figure 1). The HTH motif consists of around 20 amino acid residues distributed into two α-helices separated by a short turn, generally mediated by a Gly residue (Figure 1A). The second helix of the HTH motif (also termed “the reading head”) recognizes and binds to the target DNA *via* a number of hydrogen bonds and hydrophobic interactions, which occur between specific side chains of the protein and the exposed bases and thymine methyl groups within the major groove of the DNA, whereas the first helix, and sometimes a third one, helps to stabilize the structure of the motif (Brennan and Matthews, 1989). The HTH motif has been reported in a number of prokaryotic DNA repressor proteins as well as in eukaryotes (Brennan and Matthews, 1989). Examples of the existence of the HTH motif in solved structures of antitoxins include PezA (Khoo et al., 2007) as well as HigA (Schureck et al., 2014). Another example of antitoxin harboring the HTH motif is MsqA, although in this case the motif is present at the C-terminal region of the protein; the N-terminal region having a Zn-binding domain involved in the interaction with its cognate toxin MsqR (Brown et al., 2009).

**Table 1:** **Solved type II toxin-antitoxin structures grouped according to the DNA-binding domain of the antitoxins**.

**DNA-binding domain**	**Antitoxin**	**Toxin**	**Host organism**	**Toxin-antitoxin complex stoichiometries**	**References**
HTH	HipB	HipA	*Escherichia coli*	HipB_2_HipA_2_	Schumacher et al., 2009
	HipB_*SO*_	HipA_*SO*_	*Shewanella oneidensis*	(HipB_*SO*_)_2_(HipA_*SO*_)_2_	Wen et al., 2014
	MqsA	MqsR	*E. coli*	MqsR-MqsA_2_-MqsR	Brown et al., 2009, 2011
	HigA	HigB	*E. coli*	unknown^a^	Arbing et al., 2010
	HigA	HigB	*Proteus vulgaris*	HigA_2_HigB_2_	Schureck et al., 2014
	HigA2	HigB2	*Vibrio cholerae*	Unknown	Hadži et al., 2013
	PezA	PezT	*Streptococcus pneumoniae*	PezA_2_PezT_2_	Khoo et al., 2007
RHH	RelB	RelE	*E. coli*	RelB_2_RelE_2_	Bøggild et al., 2012
	RelB	RelE	*Methanococcus jannaschii*	RelB_2_RelE_2_	Francuski and Saenger, 2009
	DinJ	YafQ	*E. coli*	DinJ_2_YafQ_2_	Liang et al., 2014; Ruangprasert et al., 2014
	ParD	ParE	*E. coli* plasmid RK2	ParD_2_^b^	Oberer et al., 2007
	ParD1	ParE1	*Caulobacter crescentus*	(ParD1)_2_(ParE1)_2_	Dalton and Crosson, 2010
	CcdA	CcdB	*E. coli* F plasmid	(CcdA^37−72^)(CcdB)_2_ (CcdA^37−72^)_2_(CcdB)_2_	Madl et al., 2006; De Jonge et al., 2009
	HicB3	HicA3	*Yersinia pestis*	(HicB3)_4_(HicA3)_2_	Bibi-Triki et al., 2014
	FitA	FitB	*Neisseria gonorrhoeae*	(FitA-FitB)_4_	Mattison et al., 2006
	VapB3	VapC3	*Mycobacterium tuberculosis*	(VapB3)_2_(VapC3)_2_	Min et al., 2012
	VapB5^c^	VapC5	*M. tuberculosis*	(VapB5^53−86^)(VapC5)	Miallau et al., 2009
	VapB30	VapC30	*M. tuberculosis*	(VapB30)_2_(VapC30)_2_	Lee et al., 2015
	ω (regulator for ε-ζ, not antitoxin)		*Streptococcus pyogenes* pSM19035 tripartite TA system ω-ε-ζ	ε_2_ζ_2_ for the TA complex; ω_2_ for the regulator protein	Murayama et al., 2001; Meinhart et al., 2003
SpoVT/AbrB	MazE	MazF	*E. coli*	MazF_2_-MazE_2_-MazF_2_	Kamada et al., 2003; Loris et al., 2003; Zorzini et al., 2015
	VapB2	VapC2	*Rickettsia felis*	(VapC2)_4_(VapB2)_2_ (VapC2)_4_(VapC2)_4_	Maté et al., 2012
	VapB	VapC	*Shigella flexneri*	VapB_4_VapC_4_	Dienemann et al., 2011
Phd/YefM^e^	Phd	Doc	*E. coli* phage P1	Doc-Phd_2_-Doc	Arbing et al., 2010; Garcia-Pino et al., 2010
	YefM	YoeB	*E. coli*	YefM_2_YoeB	Kamada and Hanaoka, 2005
	YefM	YoeB	*M. tuberculosis*	unknown^d^	Kumar et al., 2008
Unknown^f^	aRelB	aRelE	*Pyrococcus horikoshii* OT3	(aRelB)_2_(aRelE)_2_	Takagi et al., 2005
Unknown^g^	VapB15	VapC15	*M. tuberculosis*	(VapB15)_2_(VapC15)_2_ (VapB15)(VapC15)_2_	Das et al., 2014

a *Structure was only available for the HigA antitoxin (Arbing et al., 2010)*.

b *Structure only solved for ParD in solution by NMR (Oberer et al., 2007)*.

c *N-terminal region of VapB5 could not be modeled but predicted to be RHH motif (Miallau et al., 2009)*.

d *TA complex possibly YefM_2_YoeB; only YefM was crystalized (Kumar et al., 2008)*.

e *YefM was found to share structural similarity with the Phd antitoxin with strong conservation of the N-terminal DNA-binding domain, which are thus classified as having a Phd/YefM-like fold (Arbing et al., 2010)*.

f *DNA-binding domain unclear, potentially leucine zipper dimerization with N-terminal basic residues used for DNA recognition (Takagi et al., 2005)*.

g *N-terminal residues of VapB15 could not be modeled into the electron density (Das et al., 2014)*.

The RHH proteins have been found mostly in prokaryotes. These structures are arranged as two antiparallel β-strands that generate a ribbon (Figure 1B); each strand comes from one of two protein monomers and they are involved both in dimer formation and in specific interactions with the DNA bases in the antitoxin DNA target. In the simplest form, like the transcriptional repressor protein CopG (45 residues per protomer), or the *Salmonella* phage P22 Arc repressor (53 residues per protomer) the ribbon participates in DNA recognition and in the dimerization process, so that the proteins would be mostly in a disordered state if it were a monomer. However, no monomers of the protein seem to exist, and mutational analyses indicated that dimerization and folding could be considered as part of the same process, and the proteins would only exist as dimers (Milla et al., 1995; Gomis-Rüth et al., 1998). Perhaps, and lacking further information on the structure of other antitoxins, the RHH motif seems to be the most common structural motif in antitoxins, as it is present in CcdA (Madl et al., 2006), ParD (Oberer et al., 2007), RelB (Bøggild et al., 2012), DinJ (Liang et al., 2014), FitA (Mattison et al., 2006), and VapB (Min et al., 2012).

The number of antitoxins with a SpoVT/AbrB-type domain is also steadily increasing. They all share similarities to the transcriptional regulator AbrB, found in the Gram-positive bacterium *B. subtilis* and that is involved in the regulation of many genes. The structure of the DNA-binding domain of AbrB (Figure 1C) revealed the presence of a specific domain, in which two molecules (each having two β-hairpins) dimerizes to generate a so-called layered “β-sandwich.” A similar structure has been reported for the *S. flexneri* VapBC TA pair, in which four N-terminal antitoxin VapB domains generate two DNA-binding domains; each of these domains is constructed by a three-stranded antiparallel β-sheets, and a four-stranded antiparallel β-sheet. These arrangements form a strand-switched dimer interface in which the two β-sheets are tightly packed against each other, thus generating the DNA-binding domain (Dienemann et al., 2011). Similar to VapB, but exhibiting a simpler structure is the MazE antitoxin (Kamada et al., 2003; Bobay et al., 2005), which, in turn has structural homology to the well-characterized Kis antitoxin (Kamphuis et al., 2007a,b).

The DNA binding targets of the antitoxin proteins are usually perfect or imperfect palindromic sequences (Khoo et al., 2007; Chan et al., 2011) that overlap with all or part of the promoter region; thus, binding of the antitoxin to its target would thwart the binding of the host RNA polymerase to the promoter resulting in transcription inhibition (see below).

## Autoregulation as a paradigm of type II TA loci: structure and function of the MazE antitoxin

MazEF is the first chromosomally-encoded TA discovered in *E. coli* (Aizenman et al., 1996). The *mazEF* operon is located in the *E. coli rel* locus, downstream of the *relA* gene. Expression of *mazEF* was shown to be regulated by the cellular levels of ppGpp, the product of the RelA protein. During amino acid starvation, increased levels of the alarmone guanosine tetraphosphate (ppGpp) lead to inhibition of transcription of *mazEF* and triggers programmed cell death (Aizenman et al., 1996). The MazF toxin is an endoribonuclease that cleaves cellular mRNA at the specific sequence, 5′-ACA-3′ (Zhang et al., 2003). Interestingly, MazF also cleaves ACA sites that are close to the region upstream of the AUG start site of some specific mRNAs, thus generating a pool of leaderless mRNAs. In addition, MazF also targets 16S rRNA within 30S ribosomal subunits at the decoding center, therefore removing 43 nucleotides from the 3' terminus that comprises the anti-Shine-Dalgarno. As a result, a modified translation machinery is formed to selectively translate the leaderless mRNAs to adapt to the stress condition (Vesper et al., 2011). The antitoxin MazE harbors two domains: (i) the N-terminus consists of a SpoVT/AbrB-type domain with a swapped-hairpin β-strand motif that binds to the operator to negatively autoregulate its transcription, and (ii) the C-terminal domain is intrinsically disordered and upon binding to MazF toxin will form an extended conformation that is more stable and protected from the host protease degradation (Kamada et al., 2003; Loris et al., 2003). The C-terminal tail of MazE is not directly involved in DNA binding and remained disordered upon interaction of the N-terminal domain with the DNA (Vesper et al., 2011).

Along the same operon downstream of *mazEF* is another open reading frame called *mazG*, which is co-transcribed with *mazEF*. MazG is a pyrophosphohydrolase that hydrolyses dNTPs and thus depletes ppGpp. However, MazG activity is also inhibited by the MazEF complex (Gross et al., 2006). Therefore, during amino acid starvation, in addition to inhibition of *mazEFG* transcription due to increased ppGpp, degradation of MazE will inactivate the inhibition activity of the MazEF complex against the existing MazG. Activation of MazG will deplete ppGpp levels, which in turn causes re-transcription of *mazEF* to replenish MazE, which consequently triggers the cells to emerge from their dormant state (Gross et al., 2006).

**Figure 2:**
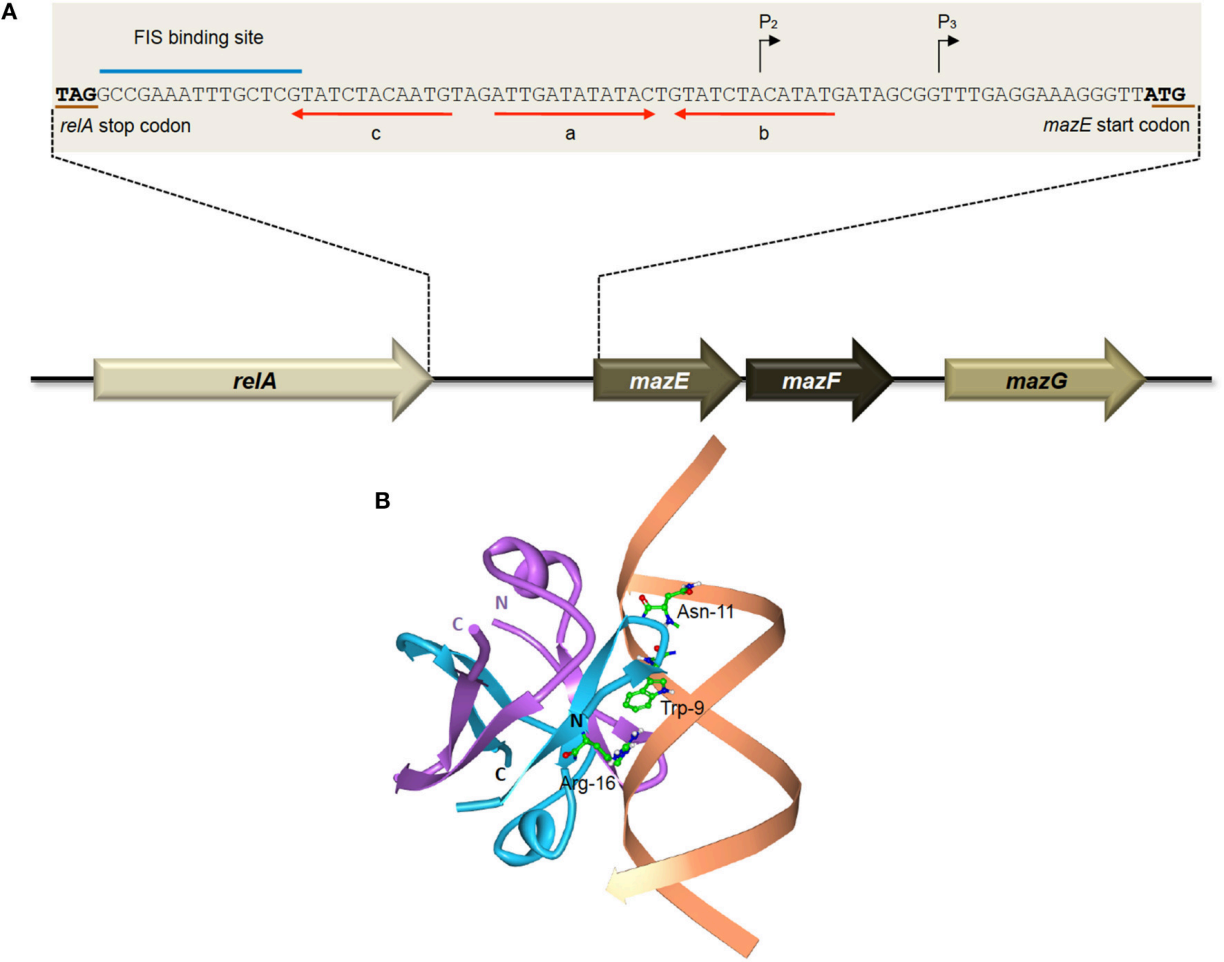
**The ***E. coli***-encoded MazEF TA system. (A)** Genetic organization of the *E. coli mazEF* operon, and the regulatory elements on the *mazEF* promoter. Black arrows denote the transcriptional start sites of promoters P2 and P3. The stop codon of RelA and the start codon of MazE are underlined in brown. The FIS binding site is indicated with a blue line. Alternating palindromic regions “c-a” or “a-b” are indicated with red arrows. Adapted and modified from Marianovsky et al. (2001). **(B)** Structure of the MazE^1−50^ antitoxin homodimer-DNA complex (PDB accession: 2MRU). The MazE^1−50^ homodimer is indicated in blue and purple with the operator DNA indicated in orange. The N- and C-termini of the two MazE^1−50^ units are as labeled. The key amino acid residues of MazE that are involved in binding to the major groove of the double-stranded “a” operator DNA, i.e., Trp-9, Asn-11, and Arg-16 (Zorzini et al., 2015), are shown for one of the MazE monomers (blue).

Two promoters, which are located 13 nucleotides apart, have been identified upstream of the *mazEFG* operon (Figure 2A). The P_2_ promoter is about 10-fold stronger than the P_3_ promoter (Marianovsky et al., 2001). Expression of both promoters is repressed by MazE and highly repressed with the MazEF complex. Within the promoters lies an unusual fragment termed the “alternating palindrome.” This alternating palindrome, which is the operator of *mazEFG*, could exist in one of two alternative states: its middle part designated “a,” complements either the downstream fragment “b” or upstream fragment “c” (Figure 2A). Binding of the MazEF complex to either arm of this alternating palindrome will strongly repress the transcription of the *mazEF* operon. The numerous mutations that were introduced into this alternating palindrome did not affect the binding efficiency of the MazEF complex, suggesting that the secondary structure of this regulatory region is more important than its DNA sequence (Marianovsky et al., 2001). MazE has higher binding affinity for fragment “a” than “b” or “c”.

Determination of the three-dimensional structure showed that the MazE homodimer binds into the major groove of DNA fragment “a,” involving the side-chains of residues Trp-9, Asn-11, and Arg-16 for the main interactions with the oligonucleotide (Figure 2B; Zorzini et al., 2015). Mobility shift assay with titration of MazF showed that MazF could increase the affinity of MazE for a single operator site where the concentration of MazE itself is not sufficient to cause a band-shift. Superposition of MazE-DNA complex on the crystal structure of the MazE-MazF complex demonstrated that the interaction between DNA and protein increased through the flanking basic regions of the MazF homodimer. This indicates that the augmentation of DNA binding by MazF is due to cooperative binding of the antitoxin and toxin to the DNA instead of an allosteric effect. However, reduced band-shift corresponding to the complex was observed after a peak with increasing MazF, and the affinity of MazE for binding to the “a” fragment is abolished at very high ratio of MazF:MazE (Zorzini et al., 2015). This resembles the conditional cooperativity phenomenon that was observed in other TA systems like *ccdAB, phd-doc*, and *relBE* whereby the expression of the TA operon is modulated by ratios of antitoxin:toxin (Overgaard et al., 2008; De Jonge et al., 2009, 2010; Garcia-Pino et al., 2010).

Besides having two promoters and an unusual alternating palindrome as operator site, the regulation of *mazEF* is also governed by another positive regulation mechanism. Further upstream of the alternating palindrome is a binding site for the factor for inversion stimulation (FIS), which positively regulates the transcription of *mazEF* operon (Marianovsky et al., 2001). FIS is a homodimer that binds and introduces bends in the DNA, thereby increasing the binding efficiency of RNA polymerase (Pan et al., 1996). The cellular level of FIS varies (up to 100-fold), depending upon the growth phase and nutritional conditions of the cells. The concentrations of FIS are highly elevated in the early exponential phase but sharply declined toward the stationary phase (Marianovsky et al., 2001), indicating positive regulation of *mazEF* is maximal at rich medium during exponential phase. Thus, the complex regulatory mechanism which combines two promoters, alternating palindromes, the FIS-binding activation site, concentrations of ppGpp and MazG, as well as the ratio and the co-operative binding activities of the MazE and MazF to the operator enables the expression of *mazEF* to become more dynamic and to ensure a prompt response to cope with various stresses or changes in the environment (Marianovsky et al., 2001).

An interesting dimension to the regulation of the MazF toxin was reported recently whereby infection of *E. coli* with bacteriophage T4 led to the addition of an ADP-ribosyl group to MazF (Alawneh et al., 2016). This chemical modification of MazF was catalyzed by phage T4-encoded Alt ADP-ribosyltransferase which transfers an ADP-ribosyl group from nicotinamide adenide dinucleotide (β-NAD^+^) to the Arg-4 residue of MazF, resulting in partial reduction of MazF cleavage activity *in vitro*. This inferred that phage T4 may harbor a unique antitoxin to inactivate MazF during T4 infection and MazF could function as an anti-phage mechanism in its *E. coli* host (Alawneh et al., 2016; Otsuka, 2016). The biological significance of the T4-dependent ADP-ribosylation of MazF and its effects on the existing *mazEF* regulatory circuit awaits further investigations.

## Regulation *via* conditional cooperativity of the *phd-doc, relBE*, and *kis-kid* loci

Phd-Doc is a TA system found on bacteriophage P1 (Lehnherr et al., 1993). The regulation of the *phd-doc* TA operon relies on the stoichiometries of the Phd antitoxin and the Doc toxin, which is a phenomenon called conditional cooperativity, as mentioned above. Like other TAs, the Phd antitoxin has an intrinsically disordered C-terminus that forms an α-helix upon binding to the Doc toxin (Garcia-Pino et al., 2008). The N-terminal domain of the Phd antitoxin is a dimerization domain that binds to the DNA operator to repress *phd-doc* expression. Doc toxin, which impedes translation by phosphorylating the conserved Thr-382 residue on elongation factor EF-Tu (Castro-Roa et al., 2013; Cruz et al., 2014), can also serve as a corepressor or derepressor depending on the molar ratio of both Doc and Phd proteins (Garcia-Pino et al., 2010). A monomeric Doc toxin has two binding sites that are able to interact with two Phd dimers, with different affinities, bridging the Phd dimers to bind more avidly to the operator. However, saturation of Doc toxin will be in favor of the high-affinity sites (H sites), outcompeting the low-affinity sites (L-sites) by Phd. This results in the restructuring of the repressor-corepressor complex to an alternative non-repressing Doc-Phd_2_-Doc complex, which cannot bind to the operator DNA due to steric reasons (Liu et al., 2008; Arbing et al., 2010; Garcia-Pino et al., 2010). Thus, the stoichiometry of Phd:Doc complex is important in modulating the regulation of *phd-doc* operon.

The *relBE* operon is one of the most prevalent and best-characterized TA system that was originally discovered on the chromosome of *E. coli* (Gotfredsen and Gerdes, 1998). The RelE toxin does not target free mRNA but cleaves mRNA in the ribosomal A site with codon specificity (Christensen and Gerdes, 2003). The RelB antitoxin neutralizes the toxic activity of RelE by displacing the α4 helix, thereby disrupting the geometry of the critical catalytic residues of the free RelE structure. RelB dimers bind to the operator through a RHH motif to autoregulate transcription (Overgaard et al., 2009). However, the affinity of RelB binding to DNA is relatively low, and addition of RelE up to a ratio of 2 RelB: 1 RelE drastically enhanced the binding affinity (Christensen-Dalsgaard et al., 2008; Overgaard et al., 2008). The RelB_2_RelE heterotrimer complexes bind strongly and cooperatively to the promoter to repress transcription. When RelE is in excess, an unusual V-shaped structure is formed, with two RelE bound at the distant ends of the RelB dimer. These heterotetramer complexes will clash when two RelB dimerization domains bind adjacently to the DNA, which leads to the complex falling-off from the operator DNA and derepressing transcription (Bøggild et al., 2012). The destabilization of the RelB_2_RelE complex from the DNA can be due to “stripping,” in which the excessive free toxin molecules invade the RelB_2_RelE heterotrimer complex; or the bulk formation of RelB_2_RelE_2_ heterotetramer complexes that sequester the heterotrimer complex (Cataudella et al., 2012). During normal cellular growth, *relB* has higher rate of translation than *relE*, leading to tenfold more RelB than RelE protein molecules (Overgaard et al., 2009). Binding of the RelB_2_RelE heterotrimer will repress transcription of *relBE* to a minimal level. When cells undergo nutritional stress, since the lifetime of RelB is 10-fold shorter than RelE (Overgaard et al., 2009), the labile RelB will be degraded more rapidly by Lon proteases and this subsequently increases the RelE:RelB ratio. Consequently, more RelB_2_RelE_2_ heterotetrameric complexes are formed that eventually derepress the repression of the *relBE* operon to replenish RelB levels in the cell. This conditional cooperativity of RelBE has also been shown to facilitate the fast recovery of cells from RelE-mediated reduction in translation when the nutritional stress is removed (Cataudella et al., 2012).

In the case of the RelBE2*Spn* operon from *S. pneumoniae*, the interaction of the two proteins with the DNA target was approached by means of band-shift, analytical ultracentrifugation, and native mass spectroscopy. The results led to the conclusion that the stoichiometry of the RelB2*Spn* antitoxin in complex with its DNA target and of the RelBE2*Spn* protein-protein complex was compatible with a heterohexamer composed of four antitoxin and two toxin protein molecules, in both conditions: protein-protein and protein-DNA complexes (Moreno-Córdoba et al., 2012).

**Figure 3:**
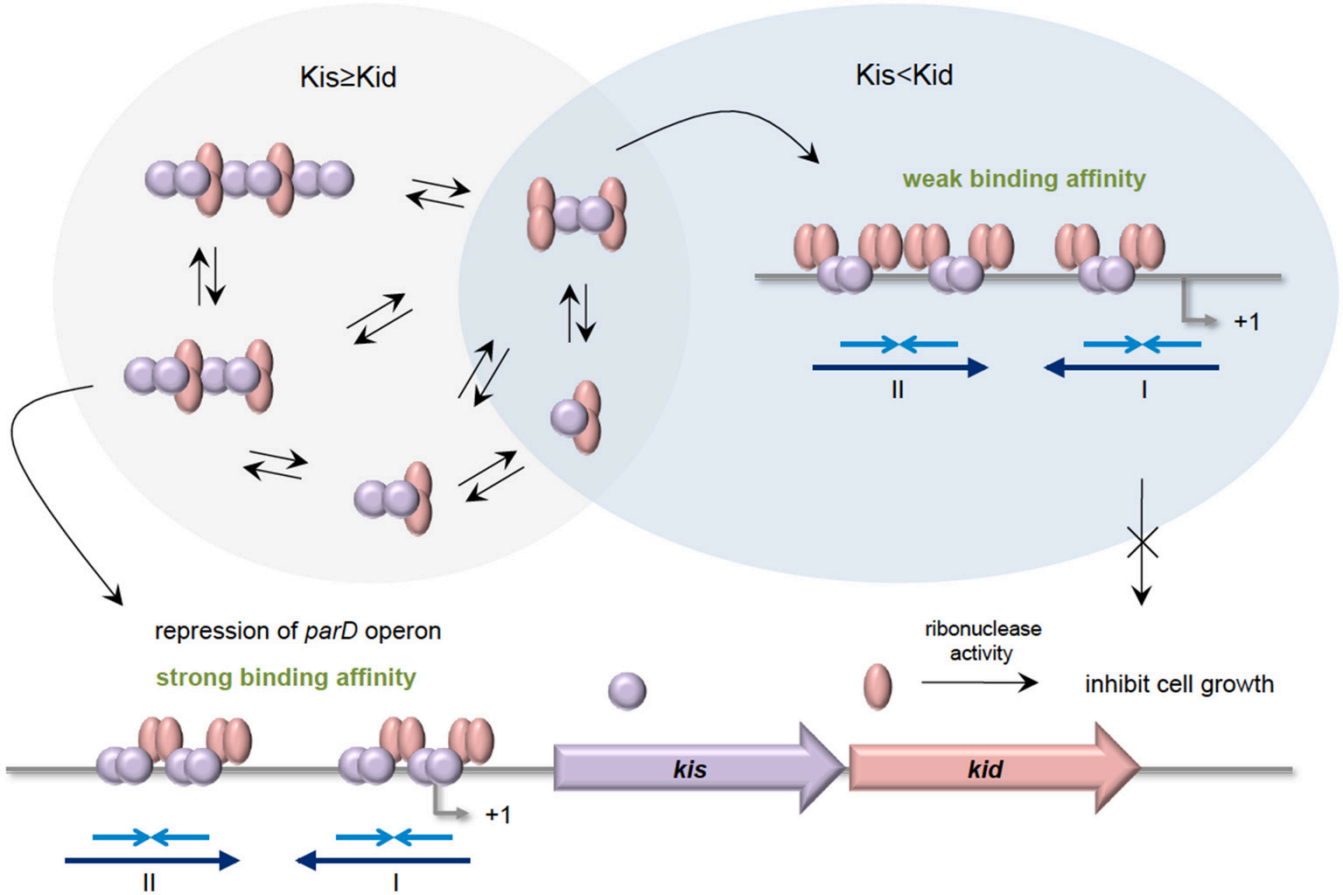
**Stoichiometries of Kis-Kid complexes and their binding affinities to ***parD*** DNA**. When the Kis antitoxin is in excess, or in equal amounts as Kid toxin, various Kis-Kid complexes are formed (e.g., [kid_2_-Kis_2_]_*n*_ or [kid_2_-Kis_2_-kid_2_-Kis_2_]_n_ etc.). The most abundant one, the Kid_2_-Kis_2_-Kid_2_-Kis_2_ octamer complex, binds strongly to the two half-sites of the *parD* DNA regions I and II with two Kis dimers, and thus strongly represses the transcription of *parD* operon. When the Kid toxin exceeds Kis antitoxin, the Kid_2_-Kis_2_-Kid_2_ hexamer is the most abundant. The Kid_2_-Kis_2_-Kid_2_ hexamer has weak affinity toward *parD* DNA as it can only bind to the two half-sites of regions I and II using one dimer. Adapted and modified from Diago-Navarro et al. (2010).

The *parD* operon of plasmid R1 from *E. coli* encodes the Kis-Kid TA (Bravo et al., 1988). Kid toxin is a ribonuclease that preferentially cleaves single stranded RNA at the 5′of the adenosine residue of sequence 5′-UA(A/C)-3′. However, cleavage at 3′of the adenosine residue on double stranded RNA was also evident (Pimentel et al., 2005; Kamphuis et al., 2006). Besides hindering the toxic effect of Kid toxin, Kis antitoxin is also a weak repressor that binds to its own promoter to regulate the transcription of *parD*. Like other typical TA, Kid toxin does not bind to the promoter but acts as a co-repressor. There are two binding regions where Kis dimers, but not monomers, preferentially bind to region I compared to region II (Figure 3). Region I harbors a perfect palindrome that overlaps the −10 consensus region of the promoter, whereas region II is an imperfect palindrome that is located upstream of the −35 sequence (Kamphuis et al., 2007b). Differential molar ratios of Kis and Kid can result in multiple complexes of Kis-Kid with different stoichiometries and oligomeric states (Monti et al., 2007). When Kid toxin is in excess, Kid_2_-Kis_2_-Kid_2_ hexamer, which has weak affinity to *parD* DNA, is most abundant. Conversely, when the Kis antitoxin equals or exceeds the concentration of the Kid toxin, strong cooperative effect will form between *parD* DNA and Kid_2_-Kis_2_-Kid_2_-Kis_2_ octamers. The Kis-Kid octameric complex can bind to the two half-sites of *parD* DNA region I and II with two dimers of the Kis antitoxin. However, the Kis-Kid hexamer can only bind to the two half-sites using one dimer, which thus explains its weak affinity (Figure 3 Kamphuis et al., 2007a,b; Monti et al., 2007; Diago-Navarro et al., 2010). Therefore, the cooperative binding between region I and region II with the Kis-Kid octamer plays an important role in the transcription regulation of the *parD* operon, and this is dependent critically on the molar ratios of Kis and Kid.

## The hybrid YefM-YoeB TA system: further complexities in TA regulation

Toxins of type II TA systems have been divided into 12 superfamilies whereas type II antitoxins have been classified into 20 superfamilies based on sequence similarities (Leplae et al., 2011). Toxins and antitoxins from different families can associate and form hybrid systems (Arbing et al., 2010; Leplae et al., 2011), with the *yefM-yoeB* locus of *E. coli* being one such example. The YefM antitoxin is from the Phd superfamily whereas the YoeB toxin belongs to the ParE/RelE superfamily; the canonical association would be Phd-Doc and RelB-RelE (Połom et al., 2013). YefM-YoeB was identified as a potential TA system based on sequence similarities of YefM with the Phd antitoxin of phage P1 (Pomerantsev et al., 2001) and homology with the *axe-txe* TA system of *Enterococcus faecium* plasmid pRUM (Grady and Hayes, 2003). Ectopic overexpression of YoeB was shown to be toxic to *E. coli* but YefM counteracted this toxicity (Grady and Hayes, 2003). Since then, the *yefM-yoeB* TA system has been found in diverse bacterial species including *S. pneumoniae* (Nieto et al., 2007; Chan et al., 2011), *Streptococcus suis* (Zheng et al., 2015), *M. tuberculosis* (Kumar et al., 2008), *Staphylococcus aureus* (Yoshizumi et al., 2009), *Staphylococcus equorum* (Nolle et al., 2013), *Lactobacillus rhamnosus* (Krügel et al., 2015), and *Streptomyces* (Sevillano et al., 2012).

**Figure 4:**
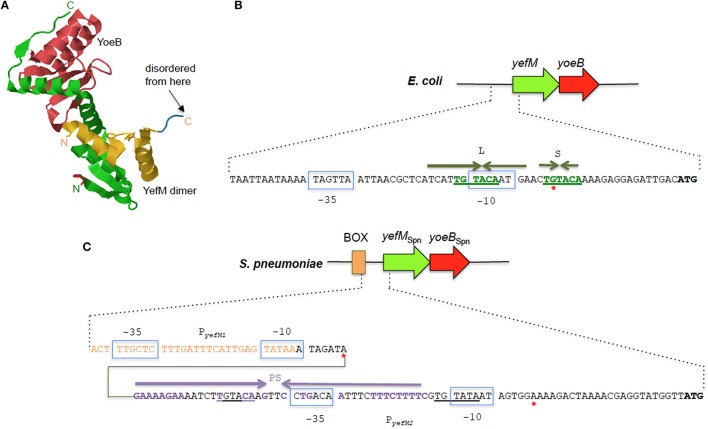
**The YefM-YoeB TA systems from ***E. coli*** and ***S. pneumoniae***. (A)** Tertiary structure of the *E. coli* YefM_2_ YoeB heterotrimeric complex (PDB accession: 2A6Q). The YefM homodimer is indicated in yellow and green with the monomeric unit containing the disordered C-termini in yellow whilst the other unit with the ordered C-termini that binds to the YoeB monomer (shown in red) is depicted in green. The N- and C-termini of the two YefM units are indicated in their respective colors. **(B)** Sequence and organization of the upstream regulatory region of the *E. coli yefM-yoeB* locus. The −10 and −35 regions of the promoter are shown in blue boxes, the core hexameric 5′-TGTACA-3′ sequence is indicated in green bold letters, and the long (L) and short (S) palindromic sequences are denoted by inverted green arrows. A red asterisk denotes the transcription start site. **(C)** Sequence and organization of the upstream regulatory region of the *S. pneumoniae yefM-yoeB*_Spn_ locus. The −10 and −35 regions of the two promoters P_*yefM*1_ and P_*yefM*2_ are shown in blue boxes. The imperfect palindrome sequence, PS, that is the operator site for P_*yefM*2_ is indicated by inverted purple arrows whereas the hexameric 5′-TGTACA-3′ sequence is underlined. Sequences that are part of the BOX element are depicted in orange letters. Red asterisks denote the transcription start sites from P_*yefM*1_ and P_*yefM*2_.

The *E. coli-*encoded YoeB toxin binds with the 70S ribosome with both the 30S and 50S subunits participating in YoeB binding and cleaves mRNA at the second position of the A site codon, thus inhibiting translation initiation in *E. coli* (Kamada and Hanaoka, 2005; Feng et al., 2013). YefM-YoeB forms a heterotrimeric YefM_2_-YoeB complex where one C-terminal peptide of the YefM dimer binds with YoeB while the other projects into the solvent (Figure 4A). The YefM dimer has symmetrical N-terminal globular structure while the C-terminus of YefM appears to be structurally disordered in the absence of YoeB and undergoes a disorder-to-order transition upon YoeB binding (Kamada and Hanaoka, 2005). YoeB forms a compact globular structure with structural similarities in its active site to RelE and other microbial RNases. Binding of YoeB to YefM in the heterotrimeric complex leads to conformational rearrangement of the RNase catalytic site of YoeB and direct obstruction by YefM, thus suppressing the toxicity of YoeB (Kamada and Hanaoka, 2005). The crystal structure of the YefM antitoxin from *M. tuberculosis* also indicated an ordered N-terminal domain and a very flexible C-terminal end that adopts different conformations in different monomers. This flexibility is postulated to make YefM more prone to proteolytic degradation (Kumar et al., 2008). YoeB-dependent mRNA cleavage is indeed activated by overproduction of the Lon protease in *E. coli*, suggesting that Lon is responsible for YefM degradation (Christensen et al., 2004).

Like most type II TA systems, the *E. coli yefM-yoeB* locus is transcriptionally autoregulated with YefM being the repressor and YoeB being a co-repressor that enhances the transcriptional repression (Kedzierska et al., 2007). There are no conventional DNA-binding motifs apparent in YefM but the N-terminal domains of the YefM dimer in the YefM_2_ YoeB trimeric complex display conserved basic patches below the symmetrical dimer interface and this was suggested as the primary DNA anchor for operator site binding (Kamada and Hanaoka, 2005; Bailey and Hayes, 2009). Two arginine residues within this basic patch (R10 and R31) were mutated and found to be essential for DNA binding by the YefM_2_ YoeB complex (Bailey and Hayes, 2009). Thus, a novel protein fold likely mediates operator recognition by YefM and we will have to await the elucidation of the YefM and YefM-YoeB structures bound to DNA for affirmation. The operator site in the *E. coli yefM-yoeB* locus consists of short (S) and long (L) palindromes, both of which possess a core hexameric 5′- TGTACA-3′ motif. The center-to-center distance between the L and S palindromes are 12 bp with the L palindrome overlapping the −10 promoter region (Figure 4B). YefM initially binds to the L palindrome followed by the S palindrome (Kedzierska et al., 2007). Changing the spacing between the two palindromes perturbs the cooperative binding of YefM-YoeB to the repeats whereby binding to the L repeat is maintained but binding to the S repeat is disrupted (Bailey and Hayes, 2009). The L and S palindromes appeared to be conserved in regions upstream of *yefM-yoeB* homologs from several bacterial genomes such as *Shigella boydii, Pseudomonas aeruginosa*, and *Erwinia carotovora* (Kedzierska et al., 2007) inferring that interaction of the YefM-YoeB homologs with these motifs could be a conserved mode of transcriptional autoregulation in these operons (Hayes and Kêdzierska, 2014).

However, investigations into the regulation of the *yefM-yoeB* locus in *S. pneumoniae*, designated *yefM-yoeB*_Spn_, indicated a different and more complex regulatory mechanism (Chan et al., 2011). Expression of the *yefM-yoeB*_Spn_ locus is driven by two σ^70^-type promoters 30 bp apart: P_*yefM*2_, which is closer to the *yefM-yoeB*_Spn_ genes and P_*yefM*1_, which lies further upstream to P_*yefM*2_ (Figure 4C). The hexameric 5′-TGTACA-3′ motif (Kedzierska et al., 2007) is also found within the pneumococcal *yefM-yoeB*_Spn_ promoter region with one of the motifs being part of a longer 44 bp incomplete palindrome sequence that overlapped the −35 region of P_*yefM*2_ (Figure 4C) and which was shown by footprinting experiments to be the operator site for the operon (Chan et al., 2011). P_*yefM*2_ is likely the native promoter for *yefM-yoeB*_Spn_ as its expression is autoregulated like other canonical type II TA systems, i.e., YefM_Spn_ represses transcription from P_*yefM*2_ while YoeB_Spn_ exerts further repression in complex with YefM_*Spn*_. However, P_*yefM*1_ appeared to be a constitutive, weaker promoter as compared to P_*yefM*2_ and is not regulated by YefM-YoeB_Spn_ (Chan et al., 2011). Interestingly, the P_*yefM*1_ promoter came about from insertion of a BOX element upstream of P_*yefM*2_ and more intriguingly, transcriptional activation was observed when the BOX element, P_*yefM*1_, P_*yefM*2_, and *yefM*_Spn_ were all in *cis* (but not when *yefM*_*Spn*_ was provided in *trans*), hinting at the possible involvement of other hitherto unknown *cis*-acting factors in the regulation of the *yefM-yoeB*_Spn_ locus (Chan et al., 2011). BOX elements are enigmatic sequences, considered to be potentially mobile and distributed randomly in numerous copies in the intergenic regions of pneumococci and related species. The occurrence and placement of the BOX element seems to be conserved in all *S. pneumoniae* strains that harbor *yefM-yoeB*_Spn_, suggesting its likely evolutionary importance to the biological function of *yefM-yoeB*_Spn_ in pneumococci (Chan et al., 2011, 2012).

A BOX-like element was also found upstream of the *yefM-yoeB*_Lrh_ locus of *L. rhamnosus* but unlike in *S. pneumoniae*, this insertion did not lead to the creation of an additional promoter (Krügel et al., 2015). Nevertheless, the regulation of the *yefM-yoeB*_Lrh_ locus appeared to be complex as well with two transcription start sites detected within the *yefM*_Lrh_ gene besides the main transcript that is expressed from a σ^70^-type promoter upstream of *yefM*_Lrh_. Furthermore, the expression levels of *yefM*_Lrh_ and *yoeB*_*Lrh*_ differed during various stages of growth and environmental stresses and appeared to respond differently in different *L. rhamnosus* strains (Krügel et al., 2015). The surprising discovery of a short transcript that is divergently transcribed and overlaps the *yoeB*_Lrh_ gene and with similarities to several type I antitoxins also hints at further complexity of the *yefM-yoeB*_Lrh_ operon regulation in *L. rhamnosus* (Krügel et al., 2015).

Such complex and multilayered regulatory control was also observed for the *yefM-yoeB* homolog, *axe-txe*, found in the *E. faecium* plasmid pRUM (Boss et al., 2013). The main promoter, *p*_at_, is autoregulated like other type II TA systems with the Axe antitoxin repressing the promoter weakly and stronger repression with the Axe-Txe TA complex. However, an internal promoter within the *axe* gene also directs the expression of the downstream *txe* toxin gene and this promoter did not appear to be regulated by Axe-Txe. Nevertheless, this internal promoter is crucial for *axe-txe* to function as a plasmid stabilization module, suggesting that it plays a role in setting the appropriate Axe:Txe ratio for proper functioning of the system (Boss et al., 2013). The finding of a cryptic transcript that originates within the *txe* reading frame along with a putative transcription terminator-like sequence downstream of *txe* that possibly modulates production of Txe are indicative of further complexities in the regulation of the *axe-txe* operon (Boss et al., 2013; Hayes and Kêdzierska, 2014).

## The MqsA antitoxin of the *mqsRA* TA loci: an antitoxin that also regulates other genes

The MqsRA locus of *E. coli* K-12 is an unusual TA locus that differs from most canonical TA systems. The MqsR (motility quorum sensing regulator) toxin was initially identified as a regulator of motility and quorum sensing, influencing the development of biofilms by mediating the cellular response to autoinducer-2 (Ren et al., 2004; González Barrios et al., 2006). The *mqsR* gene was also significantly upregulated in persister cells and, along with its downstream gene, *mqsA*, were shown to be a type II TA system (Brown et al., 2009; Yamaguchi et al., 2009; Christensen-Dalsgaard et al., 2010; Kasari et al., 2010). MqsR is a ribosome-independent endoribonuclease that specifically cleaves mRNA at 5′-GCU-3′ and, to a lesser extent, 5′-GCA-3′ sequences (Yamaguchi et al., 2009; Christensen-Dalsgaard et al., 2010).

The MqsRA system is unique in several aspects. The *mqsR* toxin gene precedes the *mqsA* antitoxin gene, an arrangement that so far has been observed only in a few type II TA loci, namely *higBA* (Tian et al., 1996), *hicAB* (Jørgensen et al., 2009), and *rnlAB* (Koga et al., 2011). The MqsA antitoxin is larger than MqsR toxin (14.7 kDa and 11.2 kDa, respectively) whereas in canonical TA systems, the toxin is larger than the antitoxin, with the exception of HicB (Jørgensen et al., 2009). Both MqsA and MqsR are also basic proteins whereas usually, the toxin is basic while the antitoxin is acidic (Kasari et al., 2010).

**Figure 5:**
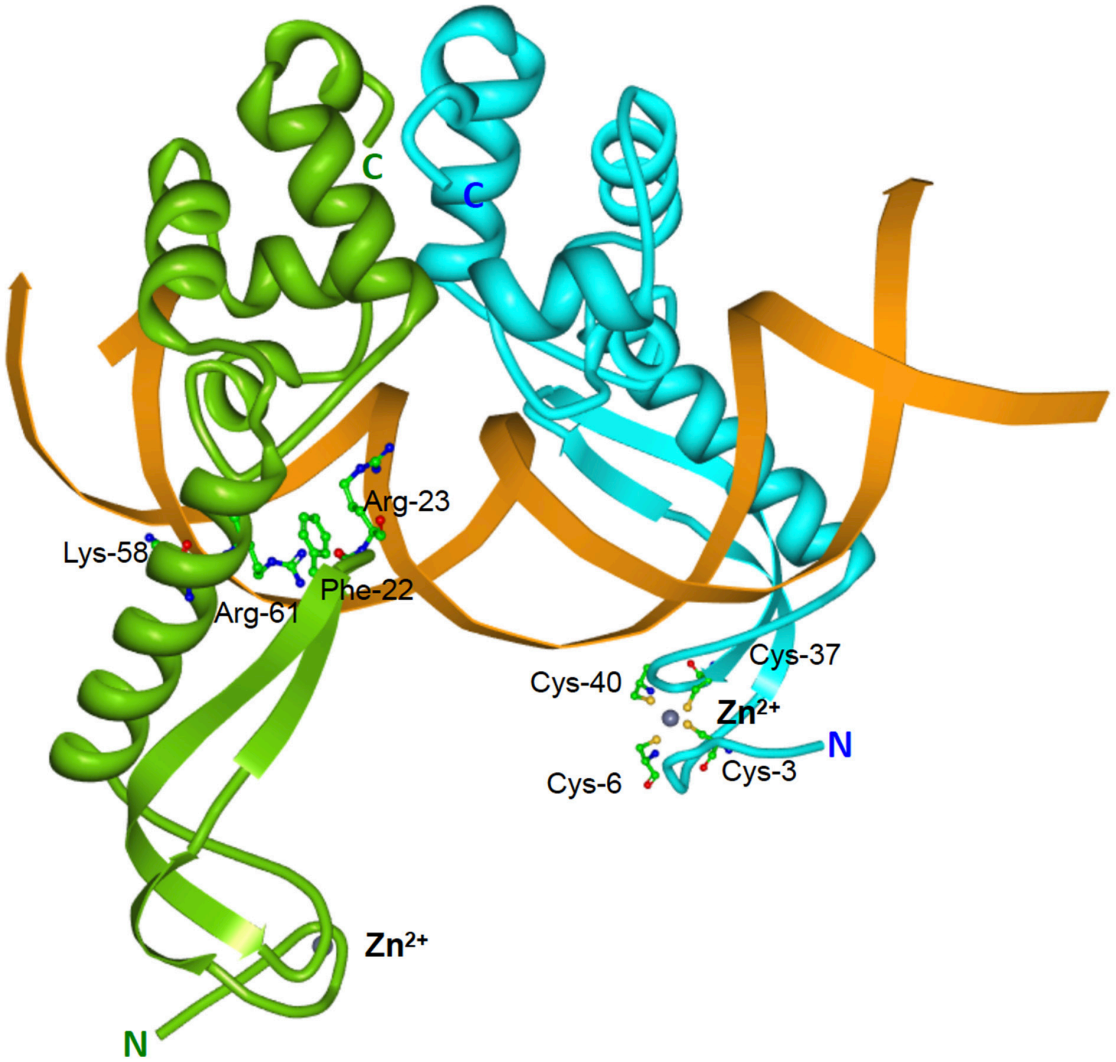
**Structure of the ***E. coli*** MqsA-DNA complex**. Tertiary structure of the *E. coli*-encoded MqsA dimer in complex with its operator DNA (PDB accession: 3O9X). The monomers of the MqsA dimer are colored either in green or in blue with their N- and C-termini indicated in their respective colors; zinc ions are shown as gray spheres; the *mqsRA* operator DNA is depicted in orange. For clarity, the MqsA amino acid residues that are crucial for interaction with operator DNA (Phe-22, Arg-23, Lys-58, and Arg-61) are shown for only one of the monomers as are the cysteine residues (Cys-3, Cys-6, Cys-37, and Cys-40) involved in coordination with the zinc ion (Brown et al., 2011).

Elucidation of the MqsRA crystal structure also revealed a few surprises. The MqsRA complex is a dimer of dimers, comprising of two copies of MqsR and two copies of MqsA (Brown et al., 2009). The MqsA antitoxin monomer is well-ordered throughout its entire length and is composed of two structurally-distinct domains connected by a flexible linker which enables the two domains to rotate independently of each other (Brown et al., 2009). The N-terminal domain of MqsA binds zinc *via* coordination with four conserved cysteine residues with the bound zinc serving as a structural and not a catalytic role (Figure 5). MqsA interacts with DNA mainly through its C-terminal HTH domain that is also responsible for MqsA dimerization (Brown et al., 2009). Most antitoxins interact with DNA through their N-terminal residues with the exception of HicB (Hayes and Kêdzierska, 2014). However, MqsA binding to DNA leads to bending of the DNA by more than 55° as well as a rotation of the N-terminal domain by more than 105°. This changes MqsA from a highly extended conformation into a narrow, elongated DNA “clamp” as a result of the formation of a DNA-binding pocket which positions several MqsA N-terminal residues (Phe-22, Arg-23, Lys-58, and Arg-61) for DNA interaction (Figure 5; Brown et al., 2011). Such conformational change is unprecedented for a bacterial antitoxin. The neutralization of MqsR toxicity by MqsA is through steric occlusion and not by direct binding of the toxin active site as in many other antitoxins. Formation of a MqsR-MqsA-DNA complex induces substantial conformational changes (Yamaguchi et al., 2009) whereby the MqsR active site residues Lys-56, Gln-68, Tyr-81, and Lys-96 face inwards and toward the other MqsR toxin pair with a separation of only 13–15 Å (Brown et al., 2011). This severely limits the accessibility of the MqsR active sites for mRNA.

Like most other type II antitoxins, the MqsA antitoxin represses transcription of *mqsRA* but instead of acting as a co-repressor, the MqsR toxin functions as a transcriptional derepressor by disrupting the MqsA-DNA interaction. In fact, a 1:1 ratio of MqsR to MqsA ablated MqsA-DNA binding due to partial overlapping of binding sites on MqsA (in particular, the Arg-61 residue) for both MqsR and DNA (Brown et al., 2013). Another unique aspect of the MqsA antitoxin is that it serves not only as a transcriptional regulator of its own *mqsRA* locus but also of several other *E. coli* genes including *mcbR, cspD, spy* and the general stress response sigma factor *rpoS* (Brown et al., 2009; Kim et al., 2010; Wang et al., 2011). An *mqsRA*-like palindromic operator site is found upstream of *rpoS* (Wang et al., 2011) and *csgD*, which encodes a master regulator of biofilm formation through the control of curli (thin proteinaceous amyloid fibers which is a major extracellular component that promotes biofilm formation) and cellulose production (Soo and Wood, 2013). CsgD also transcriptionally activates the gene for diguanylate cyclase (AdrA) which synthesizes the secondary messenger 3,5-cyclic diguanylic acid (c-di-GMP). Levels of c-di-GMP controls the switch from motility (low c-di-GMP) to sessility (high c-di-GMP) of *E. coli* (Soo and Wood, 2013). When nutrients are plentiful, MqsA increases motility by increasing the expression of *flhD*, the master regulator of *E. coli* motility partly through *rpoS* inhibition and partly through *csgD* inhibition, which also leads to low levels of c-di-GMP. Thus, in the absence of stress, MqsA functions to inhibit biofilm formation. When *E. coli* is under stressful conditions, Lon protease degrades MqsA, activating the MqsR toxin. Degradation of MqsA leads to derepression of *rpoS* and *csgD*, inhibition of *flhD*, high levels of c-di-GMP and subsequently, increased biofilm formation (Wang et al., 2011; Soo and Wood, 2013).

The MqsRA system was also recently shown to control the type V TA system, GhoST (Wang et al., 2013). The MqsR toxin enriches the *ghoT* toxin mRNA as the transcript lacks the MqsR cleavage site, 5′-GCU. GhoT functions as a membrane toxin that produces the phenotype known as ghost cells (lysed cells with damaged membranes; Wang et al., 2012). Under stressful conditions, MqsR is freed and the toxin degrades mRNAs primarily at 5′-GCU sites suc h as the 5′-end of the *ghoST* mRNA within the *ghoS* antitoxin coding sequence (which contains three 5′-GCU sites) but not *ghoT*. This leads to higher levels of GhoT toxin, which exerts its effects on the cell membrane, ultimately increasing persistence (Wang et al., 2013). Thus, there appears to be a hierarchy of TA systems in *E. coli* cells in which MqsRA controls GhoST.

## Transcriptional activators that function as antitoxins: the tale of the MrpC regulator and the solitary MazF toxin of *Myxococcus xanthus*

*Myxococcus xanthus* is a Gram-negative, rod-shaped bacterium that provides a prokaryotic model for multicellular developmental processes. Under nutrient starvation conditions, cells form aggregates which mature into fruiting bodies with some cells differentiating into spores. Other cells remain outside the fruiting bodies as persister-like cells termed peripheral rods whereas the majority of cells lyse during this developmental process (Nariya and Inouye, 2008; Lee et al., 2012; Robinson et al., 2014). The *M. xanthus* genome was found to encode a solitary *mazF* toxin gene, *mazF-mx*, without a cognate *mazE*-like antitoxin gene (Nariya and Inouye, 2008). The *mazF-mx* gene was found to be developmentally regulated and deletion of *mazF-mx* in *M. xanthus* DZF1 reduced developmental cell lysis, produced a severe delay in aggregation and reduced sporulation. Interestingly, it was reported that MrpC, which is an essential developmental transcription factor, was found to regulate the expression of *mazF-mx* and also functions as an antitoxin for MazF-mx by forming a stable complex with MazF-mx (Nariya and Inouye, 2008). The *mrpC* gene is encoded 4.44 Mbp downstream from *mazF-mx* and activates expression of many development-specific genes with strains lacking *mrpC* failing to develop and sporulate. Severe cell toxicity by MazF-mx was observed in a Δ*mrpC* mutant when *mazF-mx* expression was induced (Nariya and Inouye, 2008). It was thus proposed that the orphan *mazF-mx* in *M. xanthus* was successfully integrated into the cellular developmental programme with another transcription factor unrelated to the common cognate MazE antitoxin functioning as the surrogate antitoxin.

However, some apparently conflicting data have recently emerged regarding the MazF-mx function. Lee et al. (2012) showed that deletion of *mazF-mx* from the wild-type strains DK1622 and DZ2 had minimal to no effect on developmental cell lysis and sporulation as opposed to its deletion in strain DZF1 as reported by Nariya and Inouye (2008). It was postulated that the DZF1 background contains a *pilQ1* allele bearing two missense mutations in *pilQ* (G741S and N762G) which greatly sensitizes *M. xanthus* cells and render them more susceptible to lysis (Lee et al., 2012). Indeed, it was shown that the phenotypic effects of *mazF-mx* removal in DZF1 were recreated in strain DK1622 by introducing the *pilQ1* mutation into a Δ*mazF* mutant (Boynton et al., 2013). Lee et al. (2012) proposed the existence of two parallel, redundant pathways of developmental programmed cell death in DK1622 and DZ2, one of which is controlled by MazF-mx, and the other by an unknown mechanism which was disrupted in strain DZF1. However, Boynton et al. (2013) raised the possibility that the observed phenotypic differences may be artifactual, resulting from increased membrane permeability due to the *pilQ1* allele. Further, Boynton et al. (2013) reported that MrpC enhanced MazF-mx endoribonuclease activity in direct contrast to the inhibitory antitoxin behavior reported by Nariya and Inouye (2008) leading to a model in which MazF-mx was postulated to function without an antitoxin partner. Thus, MazF-mx seems to have elicited a scientific conundrum reminiscent of the cell death vs. cell stasis debate that erupted for the *E. coli*-encoded MazEF system more than a decade ago (Christensen et al., 2003; Gerdes et al., 2005; Engelberg-Kulka et al., 2006; Kolodkin-Gal et al., 2007; Van Melderen and Saavedra De Bast, 2009; Van Melderen, 2010). We await further experimental evidences and scientific arguments that will be presented regarding this topic.

## Tripartite type II TA loci

**Figure 6:**
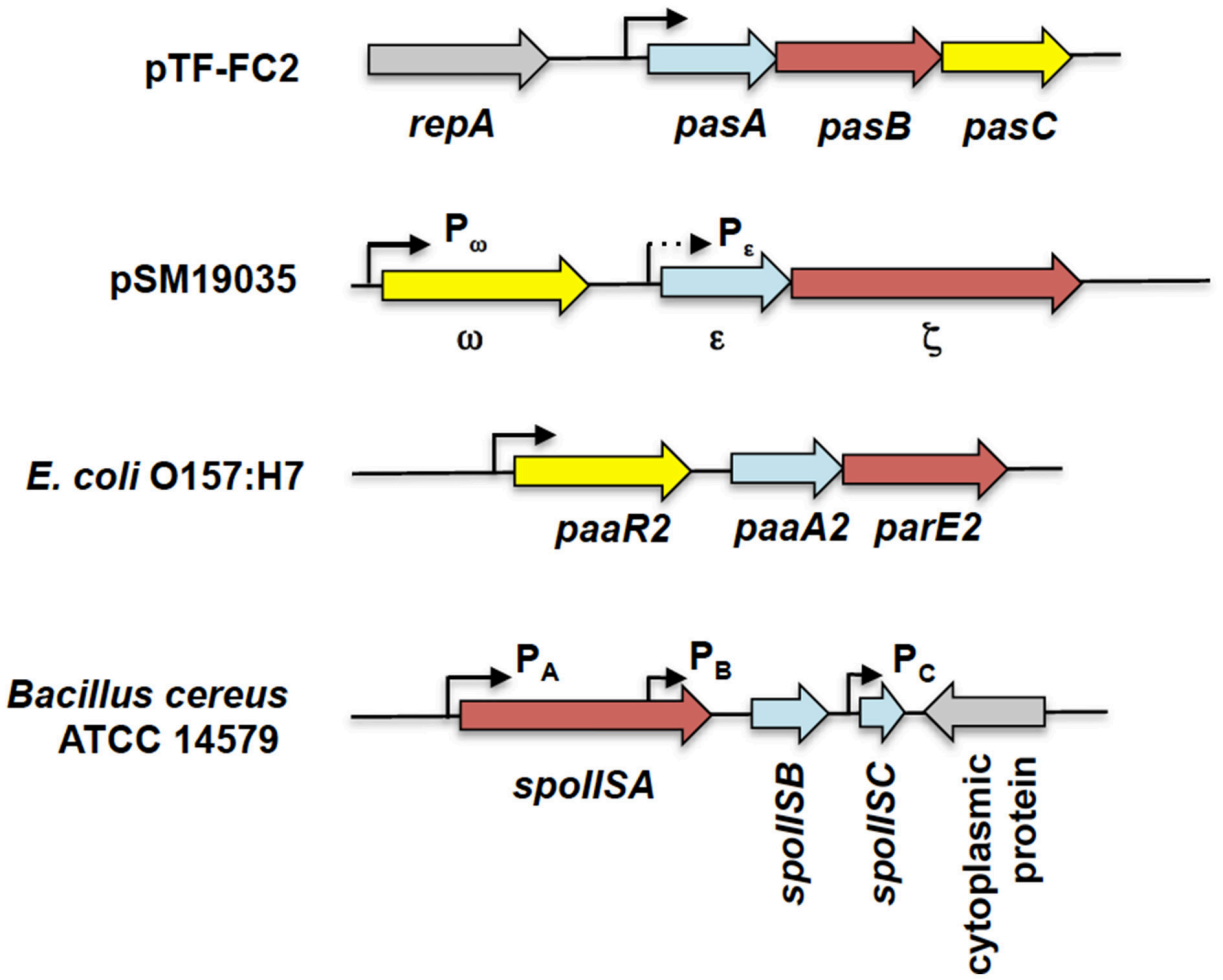
**Genetic organization of tripartite type II TA systems**. The tripartite TA systems depicted here are the *pasABC* TA system of plasmid pTF-FC2 from *A. ferroxidans* (Smith and Rawlings, 1997), the ω-ε-ζ TA system of plasmid pSM19035 from *S. pyogenes* (Cegłowski et al., 1993), the *paaR2-paaA2-parE2* system from *E. coli* O157:H7 (Hallez et al., 2010), and the *spoIIS* TA system from *B. cereus* (Melničáková et al., 2015). The antitoxin gene is depicted as blue arrows, the toxin gene as red arrows, the regulatory or third component gene as yellow arrows while gray arrows are for surrounding non-TA genes. Black line arrows indicate the relevant promoters for each TA system with the weaker P_ε_ promoter (in comparison to the P_ω_ promoter) shown as a dotted arrow. Note that the diagram is not drawn to scale.

The *pas* (plasmid addiction system) found in the 12.2 kb broad-host range, mobilizable plasmid pTF-FC2 from *Acidithiobacillus ferroxidans* (formerly *Thiobacillus ferroxidans*) was a curious example of a type II TA system with three components, the PasA antitoxin, the PasB toxin, and PasC (Figure 6), a third component that appeared to enhance the ability of the PasA antitoxin to neutralize the PasB toxin (Smith and Rawlings, 1997, 1998b; Rawlings, 1999). The *pas* locus is autoregulated with PasA as the transcriptional repressor and PasB as the co-repressor. Full repression of the *pas* promoter was observed with the PasAB complex whereas PasC did not appear to play any regulatory role (Smith and Rawlings, 1998a). A similar plasmid, pTC-F14 from *Acidithiobacillus caldus*, harbors only *pasA* and *pasB* and it was found that the two-component *pasAB* from pTC-F14 was less efficient at stabilizing a heterologous, low-copy tester plasmid pOU82 in *E. coli* when compared to the three-component *pasABC* of pTF-FC2 (Deane and Rawlings, 2004). Perhaps PasC forms a complex along with PasAB to augment the PasA antitoxin in neutralizing PasB. PasC can indeed be expressed along with PasA and PasB in *E. coli* (Smith and Rawlings, 1997) but there has yet to be any published reports on whether such a PasABC protein complex does occur. Hence, the actual function of PasC and how it helps PasA to abrogate the lethality of PasB remains unknown in the absence of further experimental results.

Another tripartite type II TA system, the ω-ε-ζ system that was discovered encoded on the low-copy number plasmid pSM19035 from a clinical isolate of *Streptococcus pyogenes* (Figure 6), differed from the *pas* locus in which the regulatory role is played by a third party. In this TA system, both the ε antitoxin and the ζ toxin have no roles in transcriptional regulation, the function which is played instead by the ω regulator (de la Hoz et al., 2000; Volante et al., 2014). ε-ζ is a type II TA system in which the 10 kDa ε antitoxin inactivates the 32 kDa ζ toxin through steric occlusion. The crystal structure of the ε-ζ complex indicated a heterotetrameric ε_2_ζ_2_ arrangement whereby the N-terminal of ε sterically blocks the ATP-binding active site of ζ (Meinhart et al., 2003). The mechanism of ζ toxicity is unique among bacterial TA toxins whereby its target is the cell wall precursor UDP-*N*-acetylglucosamine (UNAG) which is phosphorylated by ζ to UDP-*N*-acetylglucosamine-3′-phosphate (UNAG-3P) using ATP. UNAG is a basic unit of the peptidoglycan scaffold and phosphorylation of UNAG by ζ converts it into a metabolite unusable for peptidoglycan synthesis (Mutschler et al., 2011). Besides that, UNAG-3P is also a competitive inhibitor of MurA, the enzyme that catalyzes the first step in peptidoglycan synthesis. Therefore, ζ functions to inhibit bacterial cell wall synthesis (Mutschler and Meinhart, 2011; Mutschler et al., 2011). However, a recent paper reported that expression of ζ only reduced the UNAG pool and did not totally deplete it with transient expression of ζ (120 min) reversibly inducing a dormant state that was subsequently rescued by ε expression (Lioy et al., 2012; Tabone et al., 2014). It was proposed that ζ expression induces diverse responses to cope with stress with reduction in the UNAG levels as one of these responses rather than triggering a latent suicide program by depleting the UNAG pool (Tabone et al., 2014).

The ω-ε-ζ genes form an operon with two distinct promoters, *P*_ω_ upstream of the ω reading frame, and *P*_ε_, upstream of the ε reading frame (Figure 6). Transcription mainly initiates from the σ^70^-type *P*_ω_ promoter whereas *P*_ε_ appeared to be a weak, constitutive promoter that contributes marginally to transcription of the ε-ζ operon (de la Hoz et al., 2000). The ω regulator belongs to the MetJ/Arc repressor family, has an unstructured N-terminal domain followed by a RHH DNA-binding motif (Murayama et al., 2001). The binding site recognized by ω is distinctive, comprising of both palindromic and non-palidromic heptad repeats (5′-NATCACN-3′) in the operator site. A single ω_2_ dimer binds to one heptad repeat and it was suggested that cooperative binding of the ω_2_ dimer is achieved by polymerization of ω_2_ on arrays of the repeated heptad elements (Weihofen et al., 2006). ω also functions as a global regulator for plasmid pSM19035, controlling the expression of genes such as the copy control gene *copS* and the plasmid partitioning gene δ, which encodes a ParA ATPase. Interestingly, ω_2_ can either activate or repress *P*_ω_ in a concentration-dependent manner with δ_2_ acting as a co-activator by increasing the half-life of the ω_2_.*P*_ω_ DNA complexes (Volante et al., 2015).

Another tripartite type II TA system, the *paaR-paaA-parE* system, was identified in the genome of *E. coli* O157:H7 (Figure 6; Hallez et al., 2010). The ParE toxin is usually associated with the ParD antitoxin (Gerdes et al., 2005). PaaA is a novel antitoxin family that is associated with the ParE toxin, and *paaA-parE* forms an operon with a third component, *paaR* that functions as a transcriptional regulator. The *paaR-paaA-parE* operon is co-transcribed from a σ^70^-type promoter upstream of *paaR* (Hallez et al., 2010). Unlike the ω-ε-ζ system in which ε-ζ did not play any role in transcriptional regulation (Mutschler and Meinhart, 2013), the PaaA antitoxin forms a complex with the ParE toxin that repress transcription from the *paaR* promoter, albeit partially. Full repression of transcription requires the PaaR regulator (Hallez et al., 2010). However, the two repressor complexes (i.e., PaaA-ParE and PaaR) probably act independently as no three-protein complexes were detected under experimental conditions (Hallez et al., 2010). Interestingly, the genome of *E. coli* O157:H7 contains two paralogous *paaR-paaA-parE* systems with the second *paaR2-paaA2-parE2* system located in a predicted prophage. Both systems apparently coexist independently as the PaaA1 antitoxin is unable to neutralize ParE2 toxicity and vice versa (Hallez et al., 2010).

A recent report regarding the SpoIIS TA system from *Bacillus cereus* revealed a curious variation of the tripartite TA system (Melničáková et al., 2015). The *spoIIS* locus was initially identified in *B. subtilis* and was then deduced to consist of two genes, the *spoIISA* toxin-coding gene and the *spoIISB* antitoxin-coding gene, i.e., a typical type II TA locus (Adler et al., 2001). However, transcriptome analysis of *B. subtilis* had indicated the presence of a third transcriptionally active region within the *spoIIS* locus designated S458 (Nicolas et al., 2012) and which has been renamed *spoIISC* (Melničáková et al., 2015). Intriguingly, it was discovered that *spoIISC* in *B. subtilis* as well as *B. cereus* coded for an antitoxin that neutralizes the toxicity of SpoIISA. In other words, the SpoIISA toxin is neutralized by two antitoxins, SpoIISB and SpoIISC (Melničáková et al., 2015). In a departure from most type II TA systems, each gene in the *spoIIS* locus is transcribed from its own promoter (Figure 6) and each promoter is apparently transcribed under different conditions. For example, in *B. subitilis* only *spoIISA* and *spoIISB* are transcribed during nutrient deprivation, whereas during ethanol stress, only the *spoIISA* is transcribed and *spoIISC* transcribed during biofilm formation (Nicolas et al., 2012; Melničáková et al., 2015). This gives a hint at the complexity of the regulation of the *spoIIS* locus that may necessitate the need for two antitoxins, each of which could antagonize the toxicity of SpoIISA. However, at this point, there is no information as to whether the expression of the *spoIIS* genes is autoregulated.

## Caveats: the EzeT and VapC-1 toxins

The ζ toxin of the tripartite ω-ε-ζ system has two types of interesting chromosomally-encoded homologs. One homolog is exemplified by the PezT toxin of the *pezAT* system of *S. pneumoniae* whereby *pezAT* is a typical type II TA system in which the antitoxin PezA also plays an autoregulatory role, unlike the ε antitoxin (Khoo et al., 2007). PezA contains an N-terminal HTH DNA-binding motif as its repressor domain, which is fused with the three-helix bundle domain that binds and inhibits the PezT toxin. In this instance, no homologs of the ω regulator is evident in the *S. pneumoniae* genome (Khoo et al., 2007) and it is clear that ω and the repressor domain of PezA have different evolutionary origins. It was postulated that *pezA* likely originated from a fusion event of an unrelated transcriptional repressor coding sequence to the 5′-end of the coding sequence of an ε ortholog (Mutschler and Meinhart, 2013). Hints of involvement of PezAT in the pathogenicity of *S. pneumoniae* and its function in the pneumococcal pathogenicity island 1 (Brown et al., 2004; Harvey et al., 2011; Mutschler and Meinhart, 2011; Chan et al., 2012) as well as a pneumococcal integrative and conjugative element (ICE; Chan et al., 2014; Iannelli et al., 2014) warrants further investigations. Another interesting ζ homolog is found in the genomes of several bacteria. These ζ homologs are much larger than either ζ or PezT and are found not associated with a corresponding ε or PezA antitoxins (Chan et al., 2012). The functionality of these solitary ζ homologs was enigmatic as overexpression of a homolog from *Acinetobacter baumannii* was reportedly non-lethal (Jurenaite et al., 2013). However, the Meinhart group in a recent report has elegantly demonstrated that one of these solitary ζ homologs in *E. coli*, designated EzeT, consisted of a toxin domain in the C-terminal and an antitoxin domain in the N-terminal in a single polypeptide chain (Rocker and Meinhart, 2015a). *E. coli* cells that expressed full-length EzeT grew normally with no UNAG-3P detected. However, in cells that expressed an EzeT variant EzeTΔN83, that had its first 83 amino acid residues from the N-terminal deleted, a strong reduction in viability was observed in parallel with increased cell permeabilization and accumulation of UNAG-3P. Co-expression of the toxin domain (EzeTΔN83) and the N-terminal antitoxin domain [EzeT(1-82)] from separate expression vectors led to similar growth profiles as for full-length EzeT, indicative of *trans*-complementation (Rocker and Meinhart, 2015a). Intriguingly, it was found that the toxicity of EzeTΔN83 was only evident at low temperatures (below 30°C) and at 37°C, EzeT was non-functional (Rocker and Meinhart, 2015a) similar to what was reported for the GraTA system of the soil bacterium *Pseudomonas putida* (Tamman et al., 2014). Whether EzeT is autoregulated like other type II TA systems is still unknown and transcription is likely initiated from a weak promoter with a conventional −10 hexamer but without a −35 element (Rocker and Meinhart, 2015a). Nevertheless, a closer examination of solitary or orphan toxin homologs is clearly needed as EzeT has been demonstrated to be likely a new type of TA system in which a *cis*-acting antitoxin is tethered to the toxin within a single polypeptide. Besides that, large, possibly multi-domain ζ-toxin homologs linked to phosphatase or peptidoglycan-binding domains have been detected along with other toxin families such as ParE, Fic/Doc, and PemK as parts of multi-domain proteins (Rocker and Meinhart, 2015b). Their characterization and biological functions await further investigations.

The VapBC TA system is by far the most numerous among TA families with many bacterial genomes containing multiple *vapBC* loci (Pandey and Gerdes, 2005; Leplae et al., 2011; Shao et al., 2011). The VapC toxins are characterized by a PIN (PilT N-terminus) domain and display similarities to several nuclease domains. VapC from enterobacteria are tRNAses that inhibit global translation by site-specific cleavage of tRNA^fMet^ between the anticodon stem and loop (Winther and Gerdes, 2011) whereas the VapC toxins from other bacterial species have different RNA target specificities (Ahidjo et al., 2011; McKenzie et al., 2012). As with other type II TAs, the VapBC complexes bind to operators in the promoter regions to autoregulate transcription (Robson et al., 2009; Winther and Gerdes, 2012). However, the *vapBC-1* locus of nontypeable *Haemophilus influenzae* showed notable differences as in stark contrast to other VapBC homologs and type II TA systems that have been described, the VapC-1 toxin possesses DNA binding activity whereas the VapB-1 antitoxin does not interact directly with DNA (Cline et al., 2012). However, VapB-1 increases the affinity of VapC-1 for DNA and confers specificity for the operator site for the VapBC complex. The *vapBC-1* locus is also regulated by the FIS which is responsible for activation of *vapBC-1* during nutrient upshifts (Cline et al., 2012). During nutrient starvation conditions, VapB-1 would be degraded by endogenous proteases, releasing active VapC-1 toxins, and facilitating entry of *H. influenzae* cells into the persister state. When conditions favor cellular growth, FIS activates *vapBC-1* transcription and displaces any bound VapC-1 on the operator site (which is unstable in the absence of VapB-1). Levels of FIS decreases during early exponential growth, and this allows the VapBC-1 complex to bind and restore transcriptional equilibrium (Cline et al., 2012).

## Conclusions and perspectives

Our knowledge on toxin-antitoxin systems has indeed come a long way since they were coined as “addiction” modules that function to ensure the stable maintenance of plasmids in the absence of selection pressure by killing off any plasmid-free daughter cells that developed following cell division. The near ubiquity of these systems in prokaryotic genomes and their wide variety reflect their myriad functions in the prokaryotic lifestyle. The antitoxins are central to the proper functioning of these TA systems and in this review, we delved in detail on how these antitoxins usually play dual roles in regulating the expression of the TA operon as well as neutralizing the lethal action of the toxins during normal cellular growth, i.e., keeping the proverbial wolves at bay. In most cases, cellular survival hinges on maintaining the balance between the amounts of toxin and its cognate antitoxin. Hence we have seen how some TA systems have evolved beyond the basic autoregulatory circuit to incorporate additional regulatory elements. Such further complexities to the regulation of TA expression are speculated to provide additional possibilities to fine-tune and optimize the production of toxin and antitoxin under diverse environmental conditions enabling the cells to better adapt to rapid fluctuations. Such rapid fluctuations may be extreme in soil-inhabiting bacteria and related environmental niches whereas bacteria that lead a relatively “comfortable” life in a host such as pneumococci in biofilms in the human nasopharynx may have to more frequently confront changes in the host immune system. As the ectopic expression of some of the bacterial TA toxin genes leads to severe growth defects and cell death, there has been increasing interest in TAs as potential targets for novel antimicrobial agents. Several strategies have been proposed and developed to enable toxin activation in pathogenic bacteria such as interfering with the TA complex formation or the transcription of the operon itself through ligands that block the interaction of the antitoxin with the operator site. These and the potential for exploiting TAs as antimicrobial agents have been recently reviewed (Alonso et al., 2007; Mutschler and Meinhart, 2011; Williams and Hergenrother, 2012; Tanouchi et al., 2013; Hayes and Kêdzierska, 2014; Chan et al., 2015). TAs have also been harnessed as tools for biotechnology and molecular biology such as in the development of positive selection plasmid vectors (Stieber et al., 2008; Unterholzner et al., 2013). The discovery of their functionality in eukaryotic cells have opened up interesting avenues for research and development including as anticancer and antiviral gene therapies, and as a containment system for genetically modified organisms (de la Cueva-Méndez et al., 2003; Chono et al., 2011; de la Cueva-Méndez and Pimentel, 2013; Shimazu et al., 2014; Wieteska et al., 2014; Chan et al., 2015; Preston et al., 2015; Yeo et al., 2016). With more TAs being discovered and characterized in the coming months and years ahead, our understanding of their variety and complexity, particularly in the regulatory circuits of these small genetic loci, will be greatly enhanced. Additional knowledge on these systems would enable novel and improved strategies for harnessing TAs for various biomedical and biotechnological applications. These serve to underline the importance and essentiality of TA systems in modulating the prokaryotic lifestyle.

## Author contributions

WTC, ME and CCY conceived, wrote, edited and approved this review.

### Conflict of interest statement

The authors declare that the research was conducted in the absence of any commercial or financial relationships that could be construed as a potential conflict of interest.

## Abbreviations

TA: toxin-antitoxin
HTH: helix-turn-helix
RHH: ribbon-helix-helix
FIS: factor for inversion stimulation.
